# Conversion of Cytochrome P450 2D6 of Human Into a FRET-Based Tool for Real-Time Monitoring of Ajmalicine in Living Cells

**DOI:** 10.3389/fbioe.2019.00375

**Published:** 2019-11-27

**Authors:** Ghazala Ambrin, Mohammad Ahmad, Abdulaziz A. Alqarawi, Abeer Hashem, Elsayed Fathi Abd_Allah, Altaf Ahmad

**Affiliations:** ^1^Department of Botany, Faculty of Life Science, Aligarh Muslim University, Aligarh, India; ^2^Department of Physics, Syracuse University, New York, NY, United States; ^3^Plant Production Department, College of Food and Agricultural Sciences, King Saud University, Riyadh, Saudi Arabia; ^4^Botany and Microbiology Department, College of Science, King Saud University, Riyadh, Saudi Arabia; ^5^Mycology and Plant Disease Survey Department, Plant Pathology Research Institute, ARC, Gaza, Egypt

**Keywords:** FRET, nanosensor, ajmalicine, CYP2D6, flux

## Abstract

Ajmalicine is naturally present in the root bark of *Catharanthus roseus* L. and *Rauvolfia serpentina* (L.) Benth ex.Kurz. It has been extensively utilized in the treatment of hypertension across the world. The increased demand, overconsumption, and low content of the alkaloid in the plants have raised the issue of the depletion of natural sources. The metabolic engineering approach has not been successful in improving the content of the ajmalicine because the metabolic regulation of this metabolite is not known. The regulation of a metabolite in the metabolic pathway requires a tool that can carry out real-time measurement of the flux of the metabolite in living system. Given this, the present study was conducted to develop a genetically encoded FRET-based nanosensor by engineering human Cytochrome P450-2D6, an ajmalicine binding protein. The Cytochrome P450-2D6 was sandwiched between two FRET fluorophores. The design of the nanosensor brings two fluorescent proteins in conjunction with the ajmalicine binding protein, such that it undergoes FRET (Fluorescence Resonance Energy Transfer) upon binding of the ligand. The nanosensor, named as FLIP-Ajn (Fluorescence Indicator Protein for Ajmalicine), was pH stable and ajmalicine specific. The affinity of the FLIP-Ajn was 582 μM. The FLIP-Ajn successfully performed real-time measurement of ajmalicine in prokaryotic (bacteria) and eukaryotic systems (yeast, animal cell line, and plant suspension culture), thereby, establishing its biocompatibility in monitoring of ajmalicine in living cells. Besides, several affinity mutants of the nanosensor were generated through mutations in the ajmalicine binding protein to increase the detection range of the nanosensor at varying physiological scales. The non-invasiveness and high spatial and temporal resolution of the tool holds a great significance in the bio-imaging of a highly compartmentalized metabolic pathway. The flux study of ajmalicine will help in identifying the regulatory steps involved in the synthesis of the alkaloids and, hence, will improve the production rate of ajmalicine from its natural sources.

## Introduction

Ajmalicine, a naturally occurring monoterpenoid indole alkaloid, is a bioactive compound well-known for its antihypertensive and antimicrobial activities (Moreno et al., 1995; Das and Satyaprakash, 2018). It is additionally utilized in relieving the obstruction of cerebral blood flow and has demonstrated its potential in treating circulatory diseases (Misra et al., 2006). Adrenergic blocking and central depressant activities of this alkaloid was evaluated by Bhargava and Borison (1957). Cytotoxic activities of ajmalicine has also been reported (Dey and De, 2012). It has been suggested earlier that ajmalicine, along with other heteroyohimbine alkaloids acts as a precursor of various oxindole alkaloids that show an array of biological activities (Stavrinides et al., 2016). The root barks of *R. serpentina* and *C. roseus*, being the significant ajmalicine sources, have been put to indiscriminate use for extensive extraction of ajmalicine due to its high demand (Zheng and Wu, 2004; Mallick et al., 2012; Fulzele and Namdeo, 2018). Also, the low alkaloidal content in the plants demands use of natural sources in larger quantities. Conjointly, the significant depletion of the natural sources has probed into the matter of environmental concern (Zafar et al., 2019). Therefore, sustainable and enhanced production through an elaborative study of the metabolic interactions involved in the synthesis of the alkaloid is required. Previous attempts to increase the productivity of ajmalicine through cell suspension cultures, shake flask cultures, hairy root cultures, elicitation techniques, and laboratory scale-up approaches have undoubtedly contributed to better yield (Schlatmann et al., 1993; ten Hoopen et al., 1994; Almagro et al., 2010). Feasibility of the advancements at a larger scale, however, faces the predicaments of prohibitive costs and labor inputs. Given the present blockades in the path of enhanced productivity of alkaloids, the fluxomic study could generously resolve the problem by defining the regulatory elements affecting flux in a metabolic pathway. The fluxomic approach toward *in vivo* monitoring of ajmalicine dynamics could be promising in the field of research to understand the complex interconnected cellular metabolism and could equitably remove the impediments from its low yield and production. An opportunity to monitor the real-time flux of ajmalicine can be offered by the FRET nanosensor developed in the present study. It promises in providing high spatial and temporal resolution in the study of flux of ajmalicine. To date, FRET nanosensors have been successfully developed for *in vivo* study of a variety of analytes like lysine (Ameen et al., 2016), glycine betaine (Ahmad et al., 2016), zinc (Mohsin et al., 2015), glucose (Fehr et al., 2003), glutamate (Okumoto et al., 2005), and ribose (Lager et al., 2003). Given the need for a tool that can perform real-time flux of the ajmalicine in living system, a FRET-based genetically encoded nanosensor has been developed in this study.

The FRET nanosensors follow a general design of sandwiching analyte specific ligand-binding protein between the donor and acceptor fluorescent proteins. The selection of the ligand-binding protein is carried out on the basis of its capability of undergoing conformational changes in the presence of the target analyte. The changed conformation of the ligand-binding protein by the binding of the target analyte resulted in the transfer of non-radiative energy from donor fluorescent protein to acceptor fluorescent protein (FRET pair), exhibiting a changed emission intensity of the fluorophores. The nanosensor employs the variable emission intensities as a measure of the change in the metabolite concentration helping in the flux study. The beauty of these genetically encoded FRET-based nanosensors is that the monitoring of the metabolites can be performed in any cell type, and for multiple times.

## Materials and Methods

### Development of Chimeric Construct

Human cytochrome P450 2D6, an ajmalicine binding protein, has been employed as the element for binding ajmalicine for the development of the nanosensor. The structure of the protein was analyzed using RCSB PDB (PDB ID−4WNT, resolution 2.6 Å), and the nucleotide sequence of the gene (CYP2D6) encoding the protein was retrieved from KEGG (KEGG Entry−1565). The cDNA of human CYP2D6 was procured from Sinobiologicals Inc. and the amplification of the gene was achieved using a set of two primers, 5'-*accggt*cccctggccgtgatagtggccatctt-3' (FP) and 5'-*ccggt*ctagcggggcacagcacaaa-3' (RP) containing *Age*I restriction sites, denoted here in italics. Further, pDH18 vector was used for amplifying the nucleotide sequences of ECFP and Venus wherein the primers were designed to add *attB*1 and *attB*2 sites at the 5' end of the ECFP and 3' end of Venus, respectively. Subsequently, *Age*I restriction sites containing CYP2D6 gene was ligated between 3' end of ECFP and 5' end of Venus, yielding ECFP_CYP2D6_Venus chimeric sequence cloned in pGEM-Teasy vector (Promega, USA). The recombinant chimeric sequence was then introduced into pRSET B vector (Novagen, Germany) by restriction digestion at *Bam*HI and *Hin*dIII sites. The developed recombinant construct pRSET-B_ECFP_CYP2D6_Venus was designated as FLIP-Ajn (Fluorescent indicator protein for ajmalicine). The cloned construct was further introduced into the expression vectors of yeast (pYES-DEST52), plant (pEarleyGate100) and animal cells (pCDNA-DEST-40) using Gateway cloning technology (Invitrogen, USA). Initially, the pRSET-B_ECFP_CYP2D6_Venus was shuttled to pDONR222 in a BP mediated recombination reaction to generate an entry clone (pDONR222_ECFP_CYP2D6_Venus). The pDONR222_ECFP_CYP2D6_Venus was further shuttled to pYES-DEST52, pEarleyGate100, and pCDNA-DEST-40 generating expression clones, pYES-DEST52_ECFP_CYP2D6_Venus, pEarleyGate100_ECFP_CYP2D6_Venus and pCDNA-DEST-40_ECFP_CYP2D6_Venus using LR-mediated reaction. For validation of the recombination constructs, sequencing analysis was adopted (Figure S1).

### Expression and Purification of Nanosensor Protein

The FLIP-Ajn construct was transformed in *Escherichia coli* BL21(DE3) cells and allowed to grow at 21°C for a period of 24 h for the expression. The growth of the cells was continued until O.D._600_ ~0.6. Subsequently, the expression was initiated through the addition of the synthetic allolactose analog—isopropyl β-D-1- thiogalactopyranoside (IPTG). The induced cells were kept in incubator shaker at 21°C for 48 h, and the flask was covered and placed in dark conditions. The culture was then centrifuged at 4,500 × g at 4°C for 15 min, and the dissolution of the pellet was performed with 20 mM Tris-Cl (pH 7.5). After that, sonication of cells was carried out to release the nanosensor protein. The nanosensor protein was separated from the cell debris through centrifugation performed at 7,800 rpm for 30 min. The supernatant was first allowed to bind with Ni-NTA agarose resin (Qiagen, Germany) for 3 h in a Petri plate and kept at 4°C. The protein was then washed twice in a Ni-NTA His tag affinity column with ice-cold buffer comprising of 20 mM Tris-Cl, pH 7.5, and 20 mM imidazole. Finally, the elution of the protein was done with 20 mM Tris-Cl, pH 7.5, and 250 mM imidazole and stored for further study.

### Characterization of Nanosensor

Characterization of nanosensor was performed using microplate reader (Synergy H1, Biotek, USA). Initially, spectral analysis of nanosensor was carried out through measurement of the emission intensities of ECFP and Venus. The ECFP was subjected to excitation of 430 nm, and emission was recorded through spectral scanning between 450 and 600 nm at an interval of 10 nm in the presence and absence of ajmalicine. After spectral analysis, pH dependence, specificity, and affinity of the nanosensor were studied. The filters/slits used for the analysis were 430 nm/20 nm for excitation of ECFP, 485 nm/20 nm for emission of ECFP and 540 nm/20 nm for emission of Venus. The eluted protein was diluted 20 times in various buffer systems viz., Tris-Cl, PBS, TBS, and MOPS at varying pH range of 5.5–7.5 for analyzing the pH dependency and the optimum buffer system for the characterization of the nanosensor. Specificity testing of the developed nanosensor was carried out by the addition of 10 mM of various CYP2D6 inhibitors/substrates to FLIP-Ajn. These compounds were reserpine, rescinnamine, vinblastine, vincristine, quinidine, serpentine, thioridazine, and quinine. The affinity of the nanosensor was carried out through titration of the nanosensor protein at different concentrations of ajmalicine. The value derived from the ratio of Venus/ECFP emission intensities (FRET ratio) upon ligand binding provided a measure of substrate concentration. The binding affinity of ajmalicine (*K*_d_ value) was determined by applying the following equation to the ligand titration curves:

S = (r – r_apo_)/ (r_sat_ – r_apo_) = [L]/(*K*_d_ + [L]), where S stands for saturation; [L] for ajmalicine concentration; r for ratio; r_apo_ represents ratio without ajmalicine and r_sat_ with ajmalicine.

### Real-Time Monitoring of Ajmalicine

#### Bacterial Cells

The developed recombinant construct pRSET-B_ECFP_CYP2D6_Venus (FLIP-Ajn) was introduced in *E. coli* BL21(DE3) cells and grown at 37°C (150 rpm) in an incubator shaker until O.D._600_ of the bacterial growth reached 0.6. The cells were then treated with 0.5 mM IPTG and kept for 24 h at 21°C with continuous shaking for the expression of the nanosensor protein. The real-time monitoring of ajmalicine in the bacterial cells was carried out by the addition of 10 mM ajmalicine to the bacterial culture placed in the multi-well plates. Ratio of emission intensities of Venus/ECFP were recorded for 30 min and data were collected at every 5 min intervals. The control experiment includes the bacterial cells without ajmalicine. The excitation filters/slits used were same as described in the previous analysis.

#### Yeast Cells

Gateway-cloned pYES-DEST_ECFP_CYP2D6_Venus was transformed in *Saccharomyces cerevisiae*/URA3 strain BY4247. The transformed yeast cells were cultured in synthetic defined (SD) medium containing dextrose (2%) as a source of carbon and galactose (1%) for induction. The expressed yeast cells were subjected to confocal microscopic analysis with Leica TCS-SPE scan head and 63x objective lens with oil immersion system. The expressed culture was provided with 10 mM ajmalicine. Ratio of emission intensities of Venus/ECFP were recorded for 10 min and data were collected at every 20 s intervals.

#### Animal Cells

The HEK-293T (human embryonic kidney) cells were cultured in Dulbecco's modified Eagle's medium containing 50 μg/ml ampicillin, 10% fetal calf serum, and CO_2_ in an incubator shaker at 37°C. Calcium phosphate-mediated transient transfection of HEK-293T cells with pCDNA_ECFP_CYP2D6_Venus was carried out in a 6-well culture plate and was allowed to express for 2 days. Washing buffer comprised of 50 mM NaCl, 1 mM MgCl_2_, 5 mM KCl, 5 mM D-glucose, and 25 mM HEPES (pH 7.2–7.4). After a gap of 2 days after transfection, the cultured cells were set for imaging analysis using a confocal microscope with 1.53 numerical aperture, cooled charge-coupled camera, and 63x oil immersion objective lens. The culture was supplemented with 10 mM ajmalicine and the images were taken. Ratio of emission intensities of Venus/ECFP were recorded for 10 min. The laser setting used for the analysis was same for both yeast and animal cells. Data were collected after an interval of 20 s and the exposure time set for acquiring images was 300 ms.

#### Plant Cells

Leaf explants were used in the study for initiating callus. The segments of *Catharanthus roseus* leaves were collected from the herbal garden of Jamia Hamdard, New Delhi. The collected explants were then washed continuously for 20 min under tap water. The explants were then treated with 0.5% cetrimide and 0.1% HgCl_2_ for 10 and 5 min, respectively. Further sequential washing with 70% alcohol and sterilized distilled water for 1 min were performed. The culturing of sterile explants was done on MS (Murashige and Skoog, 1962) medium containing 0.5 μM 2,4-D, 3% sucrose, and 0.62% agar. The pH of the medium was maintained to 5.65, and the cultured tubes were kept in a culture room at 25 ± 2°C.

*Agrobacterium tumefaceins* strain EHA105 were transformed with pEarleyGate100_ECFP_CYP2D6_Venus, and the suspension culture was initiated after inoculating a single colony of transformed *Agrobacterium* strain in LB medium. The medium was supplemented with 50 mg/ml kanamycin, and 50 mg/ml rifampicin, and the culture was maintained at 28°C for 36 h. Next, the transformation of the callus cells was performed by adding the callus lines maintained in the culture room to the agrobacterial cell suspension culture. The culture was allowed to grow at 28°C (150 rpm) until O.D._600_ 0.6. Then, the explants were dried with the help of a sterile filter paper and co-cultivated for 3 days on MS + 100 μM acetosyringone at 25 ± 2°C. After that, the explants were first washed with 500 mg/l cefotaxime and then twice with sterile distilled water. They were again allowed to dry and further shifted to a selection medium containing MS, 10 mg/l BASTA, 250 mg/l cefotaxime and 0.5 μM 2,4-D. Subsequently, cefotaxime and antibiotic were further eliminated from the media, and the culture was maintained under 16 h photoperiod at 25 ± 2°C.

Suspension culture of *Catharanthus roseus* transformed with pEarleyGate100_ECFP_CYP2D6_Venus was observed under a Leica confocal microscope with a 10x objective. The culture was subjected to fluorescence analysis, and the ratio of Venus/ECFP emission intensities was recorded after excitation at 430 nm. Imaging was performed by using separate excitation and emission filters. A 430 nm excitation filter was employed, and 485 nm and 540 nm emission filters were used for the study. LAS-AF software (Leica, Germany) was used for recording the FRET emission intensity ratio.

### Development of Affinity Mutants

To expand the physiological range of the nanosensor, the amino acids of the ligand binding pocket of ajmalicine binding protein were point mutated using a quick-change site-directed mutagenesis kit (Stratagene, USA). Point mutations were introduced at different sites. Phenylalanine at 120 position was replaced with arginine (F120R), lysine was substituted at position 213 with alanine (L213A), glycine at position 212 was replaced with glutamine (G212Q) and aspartic acid at position 301 was substituted by glycine (D301G). Expression purification and affinity analysis of the developed mutants were carried out as described in the earlier section.

## Results

### Designing and Construction of Nanosensor

**Figure 1:**
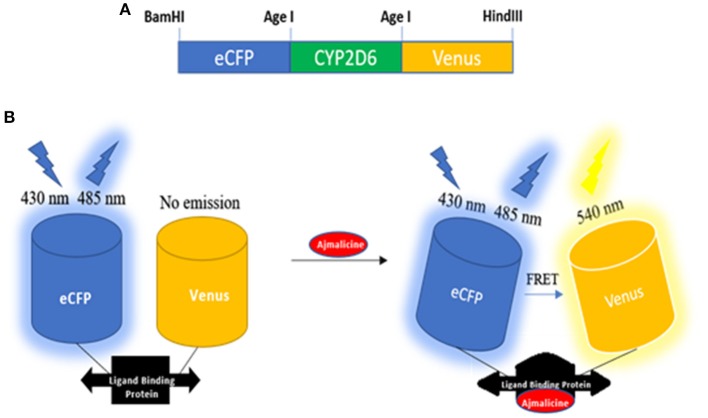
Schematic representation of the designing of nanosesnor. **(A)** The sketch of the constructed nanonanosensor. **(B)** Outline describing the working of FLIP—Ajn. FRET response observed after the introduction of ajmalicine to the system.

Cytochrome P450 2D6 was successfully converted into a sensory domain for ajmalicine. The binding of ajmalicine to the ligand binding site of the protein induced structural changes in the protein and aided in the non-radiative energy transfer between the fluorescent pair, ECFP, and Venus. The N and C terminus of CYP2D6 was joined to the fluorescent moieties (ECFP and Venus) for fulfilling the need of FRET (Figure 1). The successful development of the recombinant construct in expression vectors, including pRSET-B, was confirmed through restriction digestion as given in Figures S1–S11, and further confirmation was achieved through nucleotide sequencing analysis (Figures S1, S13).

### Spectral Analysis of Nanosensor

**Figure 2:**
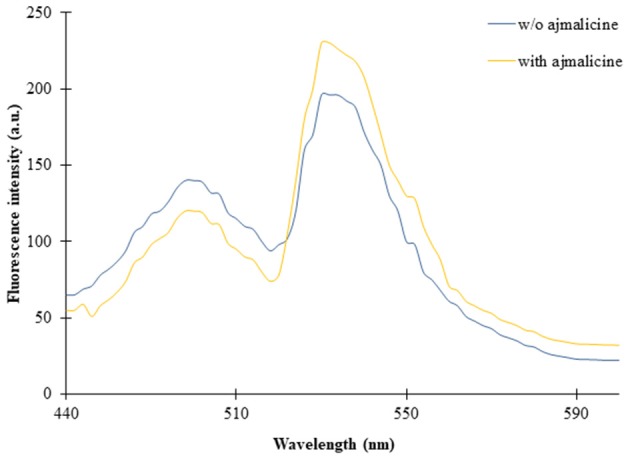
Fluorescence emission intensity recorded in a microplate reader in the absence and presence of 1 mM ajmalicine. Advancement in the fluorescence intensity of Venus is presented.

The expression of the nanosensor protein was validated through spectral analysis. An increased emission intensity of Venus was recorded in the presence of 1 mM ajmalicine (Figure 2). In the absence of ajmalicine, the emission intensity of 140 and 200 a.u. was recorded at 480 and 540 nm, respectively. Similarly, the fluorescence analysis data obtained upon addition of ajmalicine to the nanosensor protein resulted in a decreased emission intensity of 120 a.u. near 480 nm and increased fluorescence intensity of 230 a.u. near 540 nm (Figure 2). A changed Venus/ECFP emission intensities ratio in the presence and absence of ajmalicine itself implied the occurrence of FRET between ECFP and Venus.

**Figure 3:**
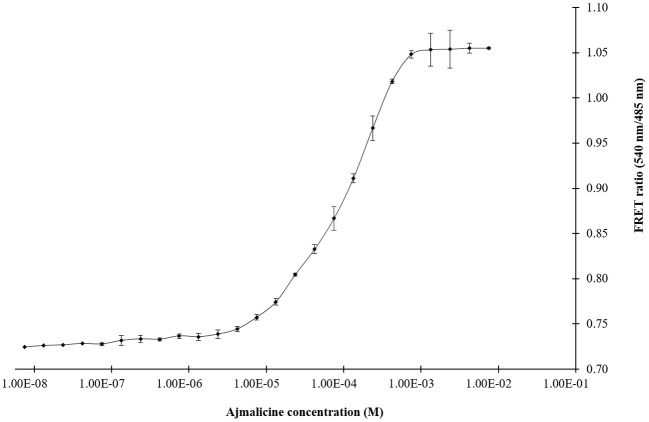
The sigmoidal graph represents the saturation of FLIP-Ajn with increasing concentration of ajmalicine. Experiment was performed with three independent replicates. Vertical bars show the standard error of the data set.

The titration experiment conducted with nanosensor protein and ajmalicine on a nanomolar to the millimolar range showed saturation of the protein with increased ajmalicine concentration. A sigmoidal curve was obtained through the data plot, where an increased FRET ratio from 0.7 to 1.05 was observed with increasing concentration of ajmalicine, and the saturation of the protein was recorded with 1 mM ajmalicine (Figure 3). The ligand-binding affinity (*K*_d_) of FLIP-Ajn was calculated as 582 μM. The concentration of the nanosensor protein was found as 0.20 mg/ml.

### pH Stability Analysis of the Nanosensor

**Figure 4:**
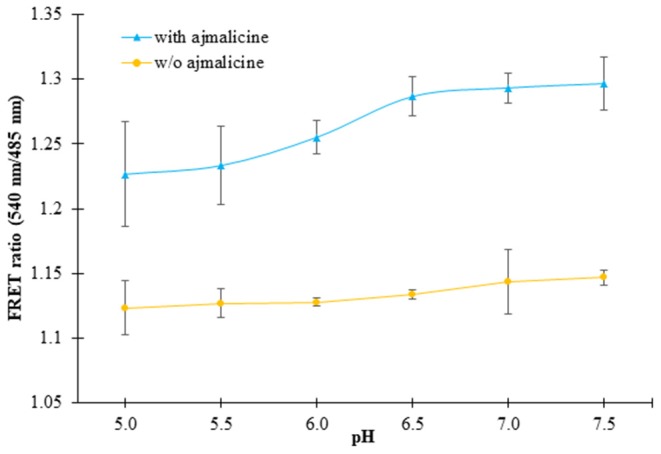
The figure shows the pH stability analysis of FLIP- Ajn. The purified protein was dissolved in different pH of PBS buffer and the change in the FRET ratio was monitored. The black and yellow line exhibits the FRET ratio determined in the presence and absence of ajmalicine (Ajn). Values plotted are the mean of three independent replicates. Vertical bars indicate the standard error.

*In vitro* pH stability analysis confirmed no hindrance of pH on the expected output of the nanosensor protein. The nanosensor protein was stable in all the buffers chosen for the analysis, viz., Tris-Cl, PBS, TBS, and MOPS. However, the best result was obtained in the buffer PBS (pH 7.0). The purified protein showed no change in FRET ratio with a reading near 1.1, in the absence of ajmalicine. However, after the addition of ajmalicine to the PBS buffer system, a notable difference in the FRET ratio was recorded. No significant change in FRET ratio was observed from pH 5.5 to pH 7.0 (Figure 4).

### Specificity Analysis of the Nanosensor

**Figure 5:**
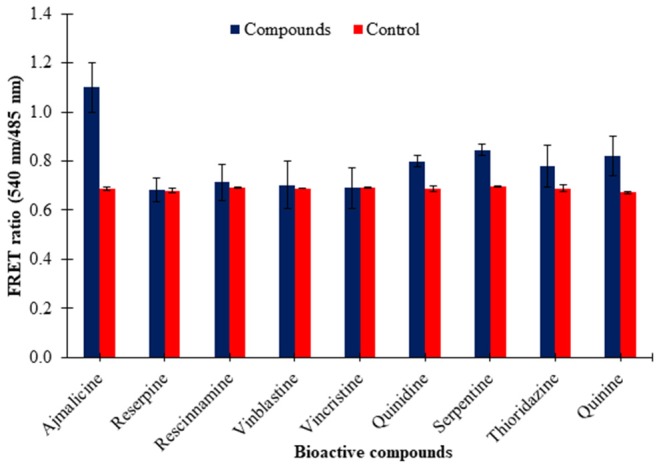
Ligand specificity analysis of FLIP-Ajn. Increased FRET ratio relative to control observed for ajmalicine alone. Study was performed with three independent replicates. Vertical bars show the standard error of the data set.

The eluted nanosensor protein subjected to specificity analysis showed a significant change in the FRET ratio in the presence of ajmalicine when compared to the control. A FRET ratio of 1.1 was recorded for the nanosensor protein by the addition of 10 mM ajmalicine. No significant change in the FRET ratio of FLIP-Ajn was found by the addition of the different CYP2D6 substrates/inhibitors (Figure 5), except in the presence of quinidine and serpentine where the FRET ratio change was ~0.12 and ~0.14 relative to control. However, it is insignificant as the FRET ratio change is <0.2. This analysis confirmed the specificity of CYP2D6 toward ajmalicine alone.

### FLIP-Ajn Mutants

**Figure 6:**
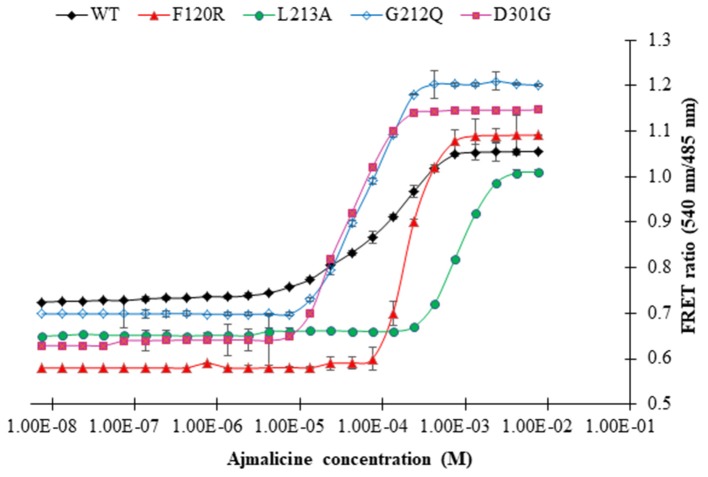
The sigmoidal graph represents the saturation of wild type nanosensor (WT) and affinity mutants of the nanosensor (F120R, L213A, G212Q, and D301G) with increasing concentration of ajmalicine. Study was performed with three independent replicates. Vertical bars show the standard error of the data set.

Four mutants of FLIP-Ajn were developed with different affinities by the help of site-directed mutagenesis. The different nanosensors have a varying binding affinity and had a broad concentration range of 24 μM-−4,500 μM (Table 1). The binding affinity (*K*_d_) for wild type (WT) nanosensor was calculated as 582 μM. Similarly, the binding constants of the mutants were also calculated. *K*_d_ values for F120R, L213A, G212Q, and D301G were calculated as 700, 3,000, 38, and 24, respectively. Maximum FRET ratio changes of 0.52 were observed in F120R (Figure 6).

**Table 1:** FLIP-Ajn affinity mutants.

**Nanosensor name^a^**	**Sequences**	**K_**d**_**	**Dynamic range^b^ (μM)**
FLIP-582 μ	Wild type	582	420–750
FLIP-3 m	L213A	3,000	1,000–4,500
FLIP-38 μ	G212Q	38	5–300
FLIP-24 μ	D301G	24	10–170
FLIP-700 μ	F120R	700	500–908

a *The nanosensor name is followed here by the determined K_d_ value*.

b *The quantification range between 10 and 90% saturation of the developed nanosensor*.

### Real-Time Flux Monitoring of Ajmalicine in Prokaryotic and Eukaryotic Systems

**Figure 7:**
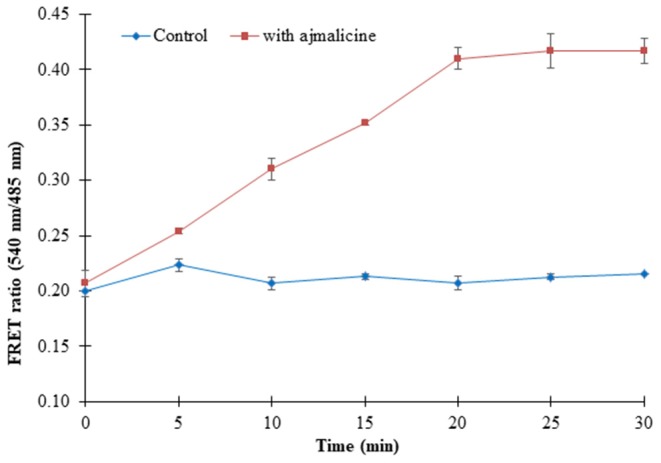
Live cell dynamics of ajmalicine in bacterial cells. The line graph represents an increase in the FRET ratio of ~0.24 upon addition of 10 mM ajmalicine. Study was performed with three independent replicates. Vertical bars show the standard error of the data set.

**Figure 8:**
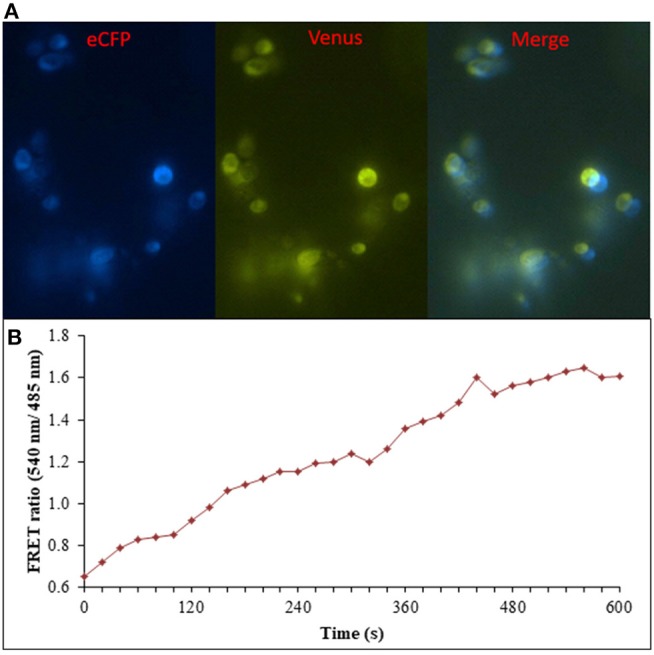
Flux monitoring of ajmalicine in yeast cells. **(A)** The confocal images of yeast cells expressing nanosensor protein. The images represent the ECFP, Venus, and merged fluorescence. **(B)** FRET emission intensities of FLIP- Ajn expressed in yeast cells. Increased FRET ratio of ~1 recorded upon addition of 10 mM ajmalicine.

**Figure 9:**
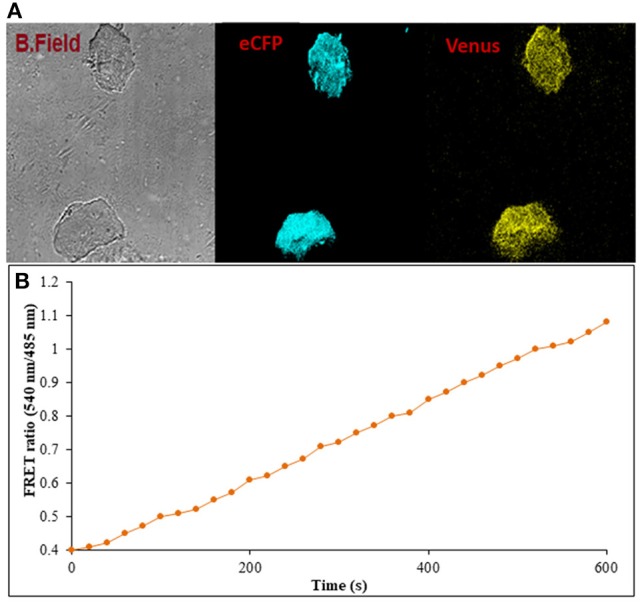
Flux monitoring of ajmalicine in mammalian cells. **(A)** The confocal imaging of FLIP-Ajn transformed mammalian cells depicting the images of bright field, ECFP, and Venus. **(B)** FRET ratio change observed in transformed HEK-293T cells. An increase in the FRET ratio from 0.4 to 1.08 recorded upon the addition of ajmalicine to the culture.

**Figure 10:**
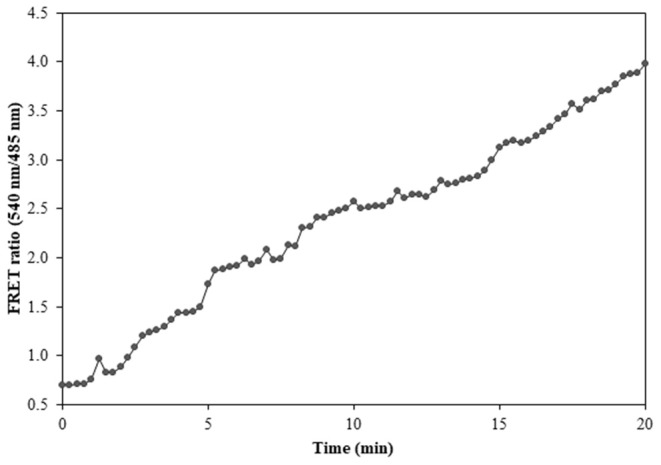
FRET ratio change in the transformed *Catharanthus roseus* cells expressing nanosensor protein. A significant increase in the emission intensity of Venus is reported within a time length of 20 min.

Prokaryotic (bacteria) and eukaryotic (yeast and animal) systems chosen for monitoring the real-time flux of ajmalicine showed an increased FRET ratio in the presence of ajmalicine. In bacterial cells, an increased FRET ratio from 0.2 to 0.4 was observed after the addition of ajmalicine externally (Figure 7). Similarly, the confocal imaging data obtained for real-time flux of ajmalicine in yeast cells showed an increase in the FRET ratio from 0.65 to 1.61 within 10 min after the addition of ajmalicine to the culture (Figure 8). The transfected HEK-293T cells also showed a changed FRET ratio of 0.4 ± 0.05 at 0 s to 0.85 ± 0.05 at 400 s upon the addition of ajmalicine, and the imaging data showed the distribution of the nanosensor protein mainly in the cytoplasm of the cultured cells (Figure 9). The *Catharanthus roseus* cells, transformed with pEarleyDate100_ECFP_CYP2D6_Venus expressed in the cytosol of the plant cells. The slow and consistent addition of ajmalicine to the suspension culture led to an increase in FRET ratio from 0.7 to 3.9 (Figure 10). No significant increase in the FRET ratio was observed in the absence of ajmalicine, but the addition of higher concentrations of ajmalicine produced data with high FRET ratio changes. The changed emission intensity ratio suggests the uptake of ajmalicine by the plant cells.

## Discussion

The pre-requisites of a genetically encoded FRET nanosensor demands conformational flexibility of the ligand binding protein, which should be sufficient to elicit energy transfer between the attached fluorophores (Fehr et al., 2002). Cytochrome P450 2D6 binds with ajmalicine leading to a change in the open structure of the protein as a result of the hydrogen bond formation of Glu-216 with ajmalicine as reported by Wang et al. (2015). The structural changes of Cytochrome P450 2D6 lattice with and without ajmalicine are depicted in the Supplementary File. The inhibitory effects of ajmalicine and serpentine on CYP2D6 were first reported by Usia et al. (2005). The working of the FRET-nanosensor has no dependency on the activity of CYP2D6 due to the inhibitory effects of ajmalicine, serpentine, and different substrates/inhibitors chosen for the study. The working of the nanosensor only requires the conformational changes of CYP2D6 upon the binding of ajmalicine, irrespective of the resultant enzymatic activity of the protein. The conformational difference brought about in the ajmalicine binding protein due to the addition of ajmalicine was sufficient to induct FRET between ECFP and Venus, as observed in Figure 2. Although thioridazine, quinidine, and quinine bring conformational changes in the CYP2D6 domain as reported by Wang et al. (2015), the FRET ratio change observed in the presence of these substrates were not significant as the FRET ratio change is <0.2 (Figure 5). The FRET occurrence is also subjected to the choice of donor/acceptor fluorophores. The fluorophore pair must have enough spectral overlap for efficient energy transfer yet have trivial differences in spectrums as to be distinguishable from each other (Held, 2005). The binding of ajmalicine to Cytochrome P450 2D6 consorted ECFP and Venus, leading to the non-radiative energy transfer between the fluorescent pair as observed by the changed emission intensities of the FRET pair. FRET ratio measurement based on the emission intensities of donor and acceptor fluorophores has been previously carried out for monitoring amino acids (Hu et al., 2017), The study further concludes no pH hindrances imposed on the affinity of nanosensor, making it suitable for monitoring the level of ajmalicine *in vivo*. The study also confirms the specificity of the nanosensor for ajmalicine alone, as supported by the significant increase in the FRET ratio of FLIP-Ajn in the presence of ajmalicine (Figure 5).

The FLIP-Ajn proved its excellent capability to monitor the level of ajmalicine in real time. The nanosensor protein successfully expressed in bacterial, yeast, animal, and plant cells, and the expected FRET ratio change observed in the studied systems validated the successful development of the nanosensor. One of the prior objectives of the developed nanosensor was to detect the metabolite dynamics with high spatial and temporal resolution in the living cells. Thoroughly, FLIP-Ajn showed its expression in the cytosol of yeast cells and the cytoplasm of animal cells, as seen in Figures 8, 9. FLIP-Ajn also expressed successfully in the cytosol of plant cells. The further addition of targeting sequences to the nanosensor protein will allow in the imaging of different sub-compartments of the cells and will enhance our ability to monitor the pathway involved in the production of ajmalicine. The targeting of the nanosensor protein to subcellular compartments has previously been achieved in a range of FRET- nanosensors, viz., FLIPglu in nuclei (Fehr et al., 2004). The generated mutants of the FRET- nanosensor also enhanced the scope of detection of the nanosensor and hence provide its usefulness in the measurement of ajmalicine levels in the broad physiological range.

## Conclusion

The study reported the development of a fluorescence indicator protein for ajmalicine through the sandwiching of CYP2D6 between a FRET pair (ECFP and Venus). The nanosensor was found pH stable ≥ 7.0, specific for ajmalicine, i.e., non-responsive to the selected inhibitors/substrates of CYP2D6 and the binding affinity of the nanosensor protein was calculated as 582 μM. The expression of the nanosensor protein was demonstrated in *E. coli* BL21 cells, *Saccharomyces cerevisiae*/URA3 strain BY4247, human embryonic kidney (HEK-293T) cells and suspension cells of *Catharanthus roseus* by fluorescence analysis in the presence of ajmalicine. The study also developed different CYP2D6 mutants through site-directed mutagenesis, yielding a set of nanosensors with varying binding affinities that would be effective for the measurement of ajmalicine levels in broad concentration ranges. FRET-based nanosensor for ajmalicine, FLIP- Ajn, proved to be a useful tool in studying the real-time flux of ajmalicine in living cells. The expression of the nanosensor in four different hosts representative of prokaryotic and eukaryotic microorganisms, as well as the animals and plants cells used in the study successfully, allowed the flux monitoring of the metabolite. The non-invasiveness and high spatial and temporal resolution of the tool holds a great significance in the bio-imaging of a highly compartmentalized metabolic pathway. The attachment of signal peptides to the nanosensor for expression in specific compartments of the plant cells will open new doors in understanding the intricate pathway operating in nature. The flux study of ajmalicine will also help in identifying the regulatory steps involved in the synthesis of the alkaloid and hence will effectively improve the production rate of ajmalicine from its natural sources.

## Data Availability Statement

All datasets generated for this study are included in the article/Supplementary Material.

## Author Contributions

GA, AA, and MA envisaged and designed the experiments. GA and AA performed the experiment. GA, AAA, and EA analyzed the data. GA, AA, AAA, AH, MA, and EA drafted the manuscript. All authors have read and approved the final manuscript.

### Conflict of Interest

The authors declare that the research was conducted in the absence of any commercial or financial relationships that could be construed as a potential conflict of interest.
