# Supplementary material for: Conversion of Cytochrome P450 2D6 of Human Into a FRET-Based Tool for Real-Time Monitoring of Ajmalicine in Living Cells
Source: Front Bioeng Biotechnol. 2019 Nov 27;7:375. doi: 10.3389/fbioe.2019.00375 (PMC6890717; doi:10.3389/fbioe.2019.00375)
Supplement: Supplementary file 1 [file Table_1.DOCX]

Nucleotide sequence of CYP2D6 NCBI Ref Seq: [NM_000106.4](https://www.ncbi.nlm.nih.gov/nuccore/NM_000106.4)

ATGGGGCTAGAAGCACTGGTGCCCCTGGCCGTGATAGTGGCCATCTTCCTGCTCCTGGTGGACCTGATGCACCGGCGCCAACGCTGGGCTGCACGCTACCCACCAGGCCCCCTGCCACTGCCCGGGCTGGGCAACCTGCTGCATGTGGACTTCCAGAACACACCATACTGCTTCGACCAGTTGCGGCGCCGCTTCGGGGACGTGTTCAGCCTGCAGCTGGCCTGGACGCCGGTGGTCGTGCTCAATGGGCTGGCGGCCGTGCGCGAGGCGCTGGTGACCCACGGCGAGGACACCGCCGACCGCCCGCCTGTGCCCATCACCCAGATCCTGGGTTTCGGGCCGCGTTCCCAAGGGGTGTTCCTGGCGCGCTATGGGCCCGCGTGGCGCGAGCAGAGGCGCTTCTCCGTCTCCACCTTGCGCAACTTGGGCCTGGGCAAGAAGTCGCTGGAGCAGTGGGTGACCGAGGAGGCCGCCTGCCTTTGTGCCGCCTTCGCCAACCACTCCGGACGCCCCTTTCGCCCCAACGGTCTCTTGGACAAAGCCGTGAGCAACGTGATCGCCTCCCTCACCTGCGGGCGCCGCTTCGAGTACGACGACCCTCGCTTCCTCAGGCTGCTGGACCTAGCTCAGGAGGGACTGAAGGAGGAGTCGGGCTTTCTGCGCGAGGTGCTGAATGCTGTCCCCGTCCTCCTGCATATCCCAGCGCTGGCTGGCAAGGTCCTACGCTTCCAAAAGGCTTTCCTGACCCAGCTGGATGAGCTGCTAACTGAGCACAGGATGACCTGGGACCCAGCCCAGCCCCCCCGAGACCTGACTGAGGCCTTCCTGGCAGAGATGGAGAAGGCCAAGGGGAACCCTGAGAGCAGCTTCAATGATGAGAACCTGTGCATAGTGGTGGCTGACCTGTTCTCTGCCGGGATGGTGACCACCTCGACCACGCTGGCCTGGGGCCTCCTGCTCATGATCCTACATCCGGATGTGCAGCGCCGTGTCCAACAGGAGATCGACGACGTGATAGGGCAGGTGCGGCGACCAGAGATGGGTGACCAGGCTCACATGCCCTACACCACTGCCGTGATTCATGAGGTGCAGCGCTTTGGGGACATCGTCCCCCTGGGTGTGACCCATATGACATCCCGTGACATCGAAGTACAGGGCTTCCGCATCCCTAAGGGAACGACACTCATCACCAACCTGTCATCGGTGCTGAAGGATGAGGCCGTCTGGGAGAAGCCCTTCCGCTTCCACCCCGAACACTTCCTGGATGCCCAGGGCCACTTTGTGAAGCCGGAGGCCTTCCTGCCTTTCTCAGCAGGCCGCCGTGCATGCCTCGGGGAGCCCCTGGCCCGCATGGAGCTCTTCCTCTTCTTCACCTCCCTGCTGCAGCACTTCAGCTTCTCGGTGCCCACTGGACAGCCCCGGCCCAGCCACCATGGTGTCTTTGCTTTCCTGGTGACCCCATCCCCCTATGAGCTTTGTGCTGTGCCCCGC

Nucleotide sequence of ECFP

ATGGTGAGCAAGGGCGAGGAGCTGTTCACCGGGGTGGTGCCCATCCTGGTCGAGCTGGACGGCGACGTAAACGGCCACAAGTTCAGCGTGTCCGGCGAGGGCGAGGGCGATGCCACCTACGGCAAGCTGACCCTGAAGTTCATCTGCACCACCGGCAAGCTGCCCGTGCCCTGGCCCACCCTCGTGACCACCCTGACCTGGGGCGTGCAGTGCTTCAGCCGCTACCCCGACCACATGAAGCAGCACGACTTCTTCAAGTCCGCCATGCCCGAAGGCTACGTCCAGGAGCGCACCATCTTCTTCAAGGACGACGGCAACTACAAGACCCGCGCCGAGGTGAAGTTCGAGGGCGACACCCTGGTGAACCGCATCGAGCTGAAGGGCATCGACTTCAAGGAGGACGGCAACATCCTGGGGCACAAGCTGGAGTACAACTACATCAGCCACAACGTCTATATCACCGCCGACAAGCAGAAGAACGGCATCAAGGCCAACTTCAAGATCCGCCACAACATCGAGGACGGCAGCGTGCAGCTCGCCGACCACTACCAGCAGAACACCCCCATCGGCGACGGCCCCGTGCTGCTGCCCGACAACCACTACCTGAGCACCCAGTCCGCCCTGAGCAAAGACCCCAACGAGAAGCGCGATCACATGGTCCTGCTGGAGTTCGTGACCGCCGCCGGGATCACTCTCGGCATGGACGAGCTGTACAAG

Nucleotide sequence of Venus

ATGGTGAGCAAGGGCGAGGAGCTGTCACCGGGGTGGTGCCCATCCTGGTCGAGCTGGACGGCGACGTAAACGGCCACAAGTTCAGCGTGTCCGGCGAGGGCGAGGGCGATGCCACCTACGGCAAGCTGACCCTGAAGCTCATCTGCACCACCGGCAAGCTGCCCGTGCCCTGGCCCACCCTCGTGACCACCCTCGGCTACGGCCTCAGTGCTTCGCCCGCTACCCCGACCACATGAAGCAGCACGACTTCTTCAAGTCCGCCATCCCGAAGGCTACGTCCAGGAGCGCACCATCTTCTTCAAGGACGACGGCAACTACAAGACCCGCGCCGAGGTGAAGTTCGAGGGCGACACCCTGGTGAACCGCATCGAGCTGAAGGGCATCGACTTCAAGGAGGACGGCAACATCCTGGGGCACAAGCTGGAGTACAACTACAACAGCCACGACTTCAAGGAGGACGGCAACATCCTGGGGCACAAGCTGGAGTACAACTACAACAGCCGCACAACATCGAGGACGGCGGCGTGCAGCTCGCCGACCACTACCAGCAGAACACCCCCATCGGCGACGGCCCCGTGCTGCTGCCCGACAACCACTACCTGAGCTACCAGTCCAAGCTGAGCAAAGACCCCAACGAGAAGCGCGATCACATGGTCCTGCTGGAGTTCGTGACCGCCGCCGG GATCACTCTCGGCATGGACGAGCTGTACAA

Fig. S1: Nucleotide sequences of the cloned CYP2D6, ECFP and Venus


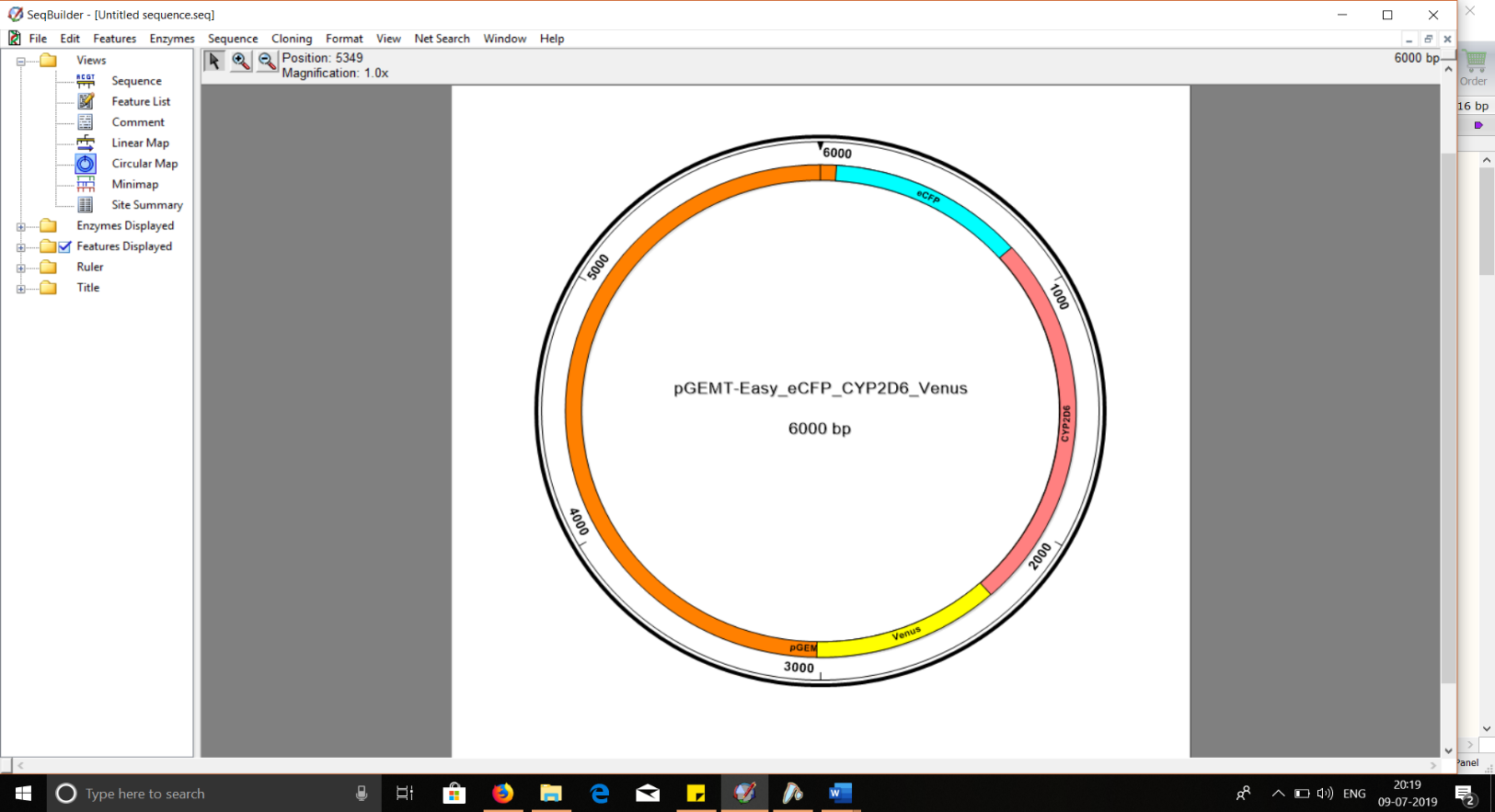


Fig. S2. Schematic representation of pGEMT-Easy_ECFP_CYP2D6_Venus


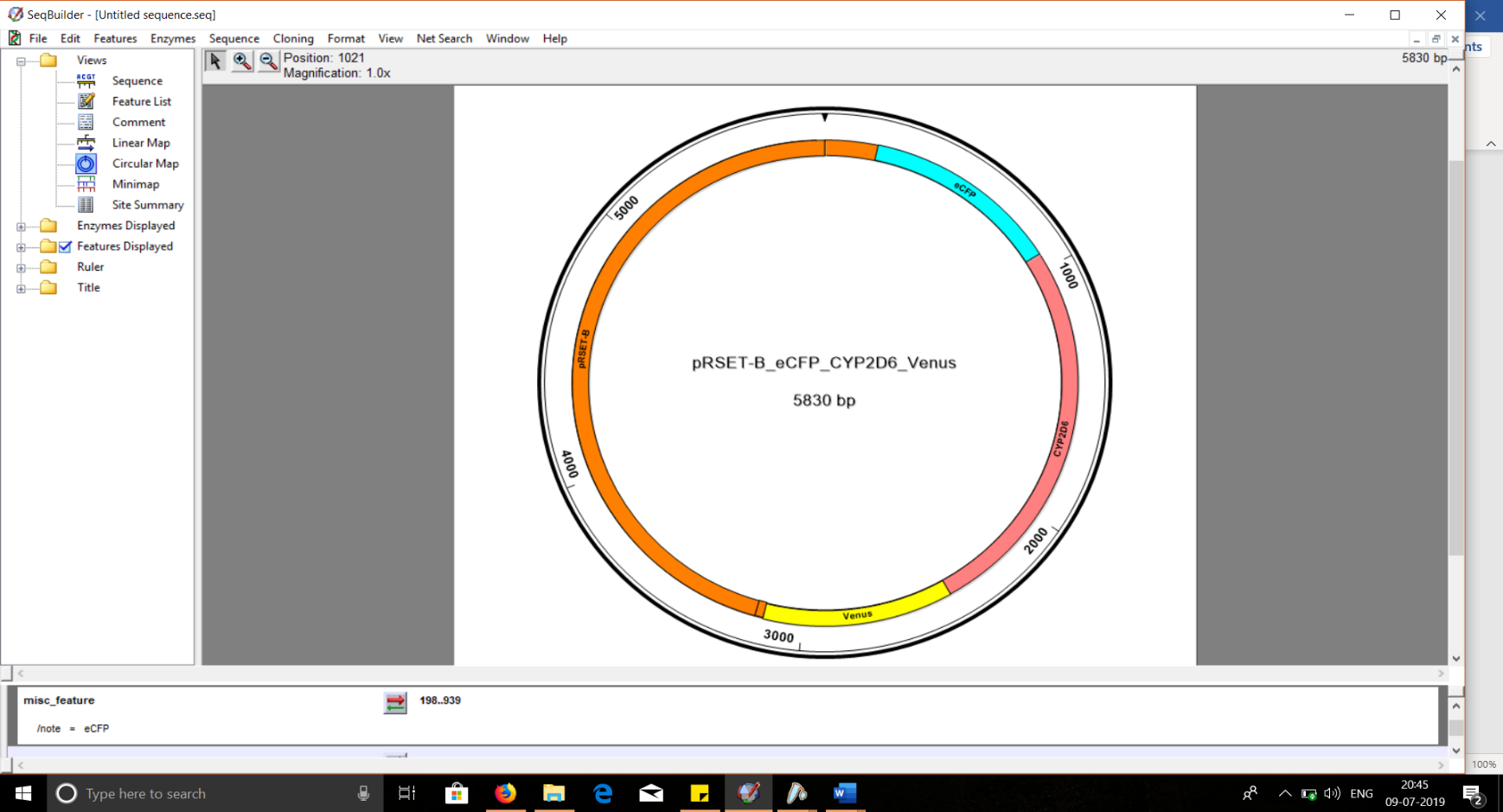


Fig. S3. Schematic representation of pRSET-B_ECFP_CYP2D6_Venus


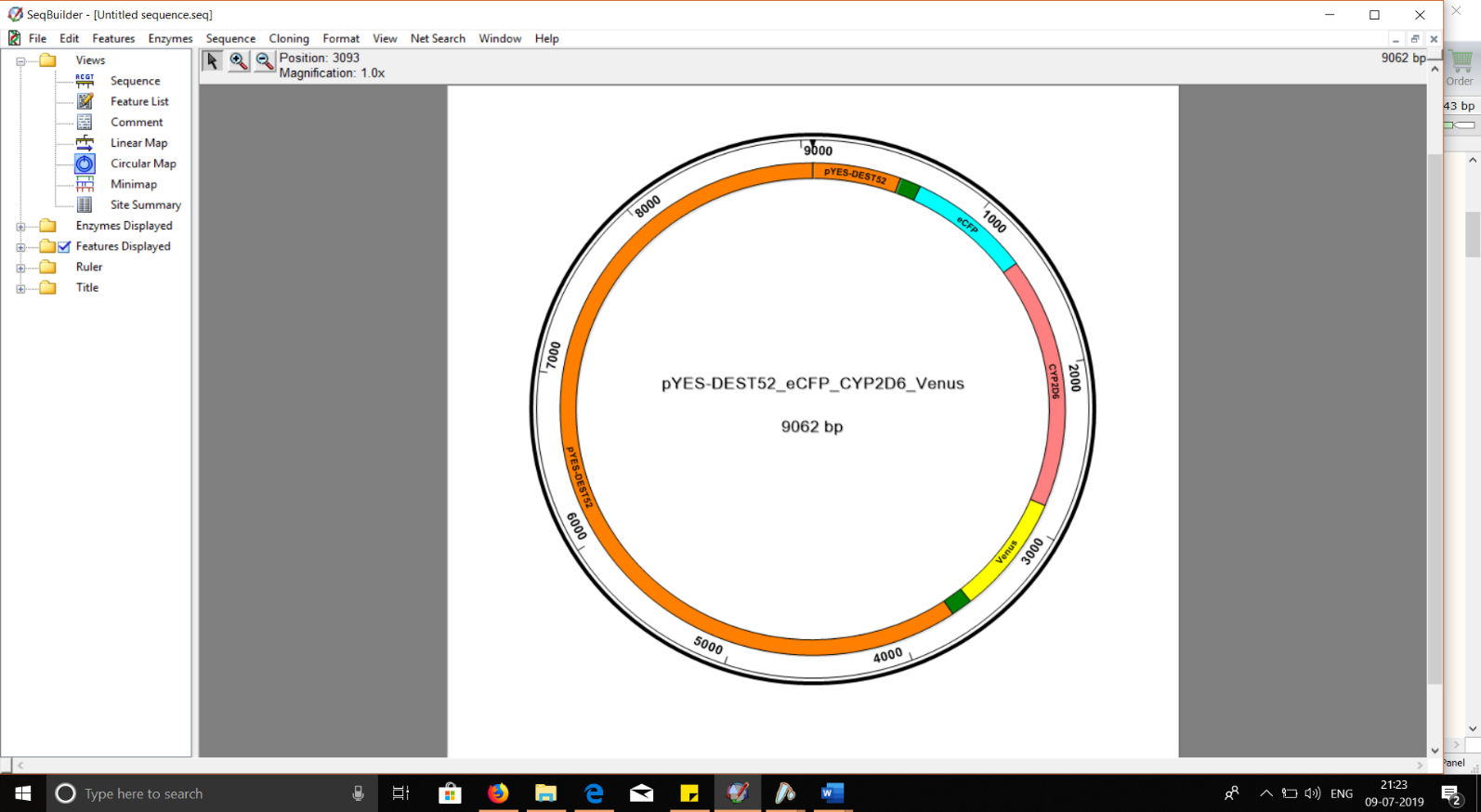


Fig. S4. Schematic representation of pYES-DEST52_ECFP_CYP2D6_Venus


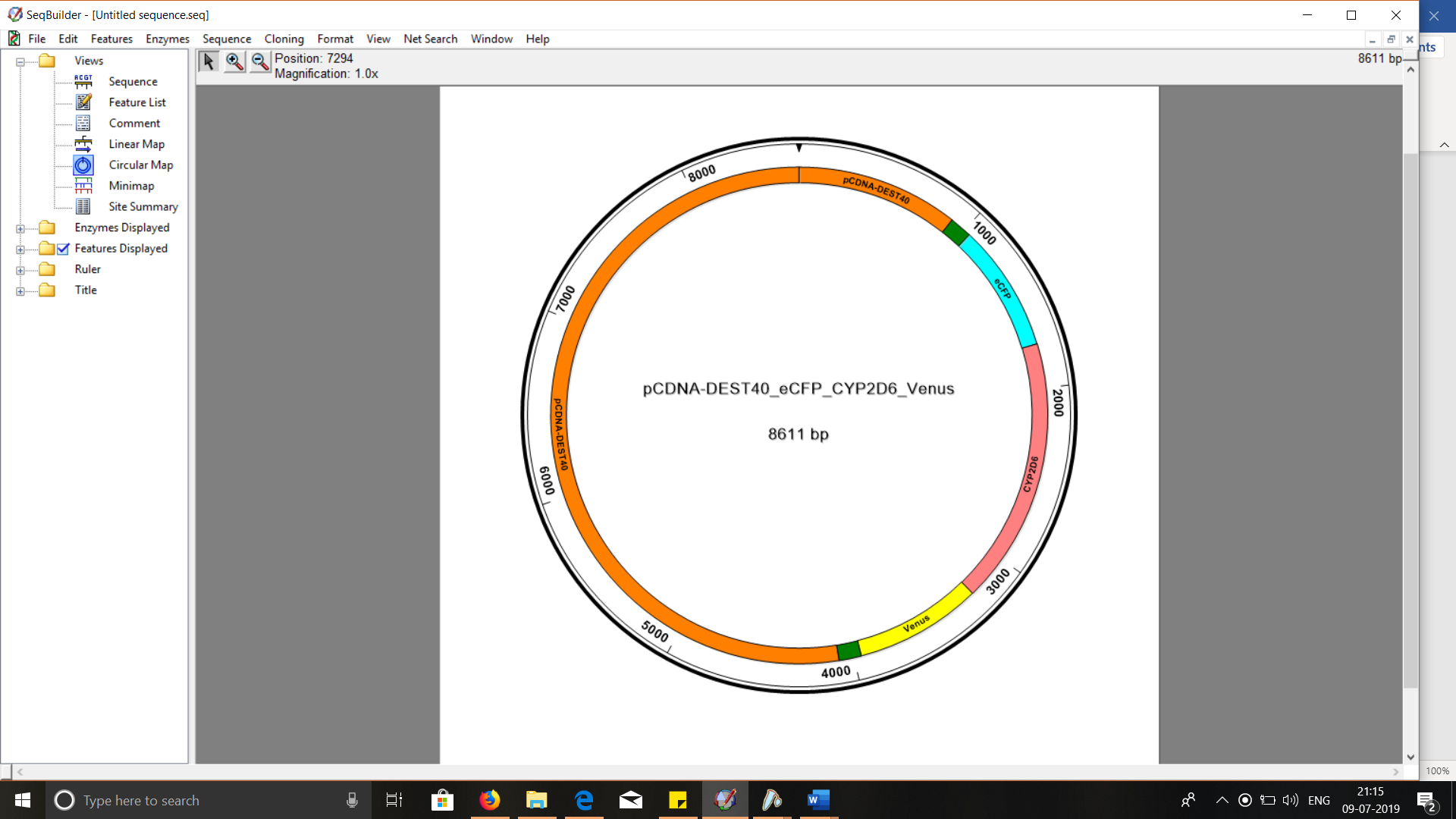


Fig. S5. Schematic representation of pCDNA-DEST40_ECFP_CYP2D6_Venus


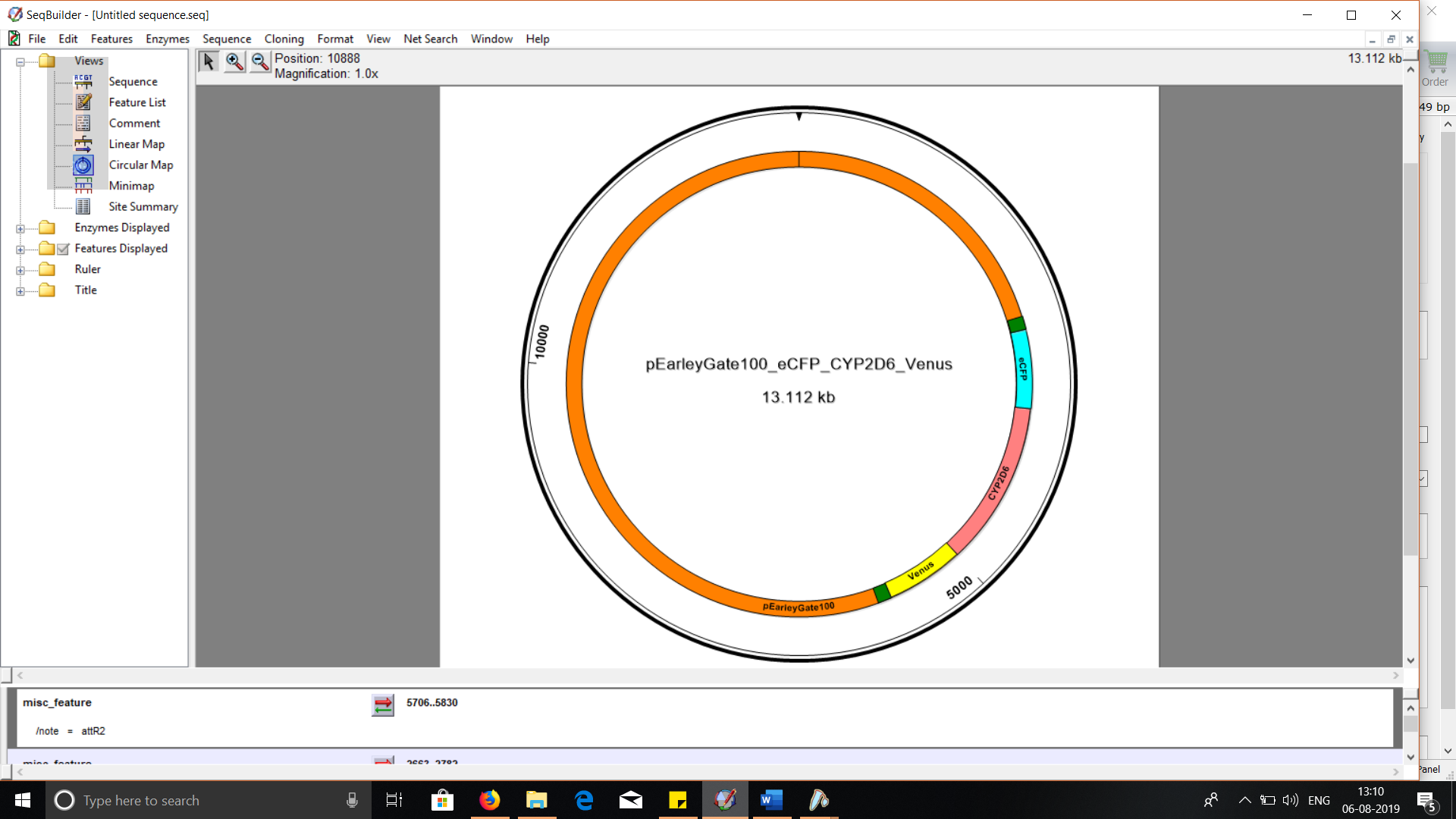


Fig. S6. Schematic representation of pEarleyGate100_ECFP_CYP2D6_Venus


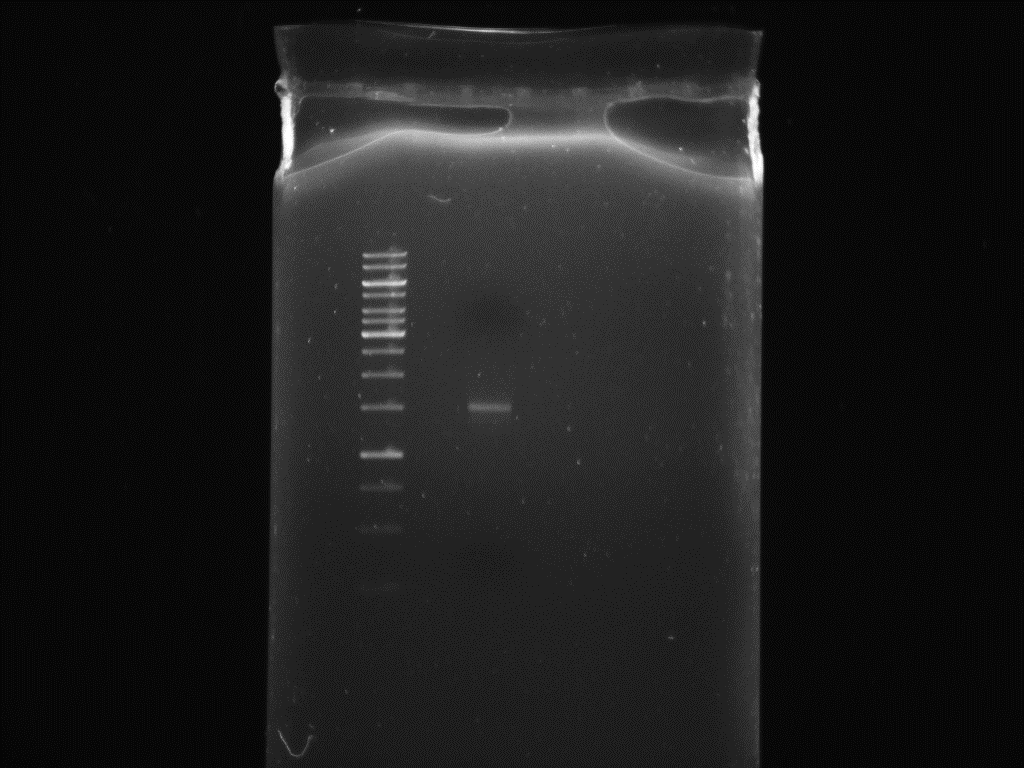


10000 bp

8000 bp

1.5 kb

1000 bp

6000 bp

Fig. S7. PCR amplified CYP2D6 gene of band size 1.5 kb


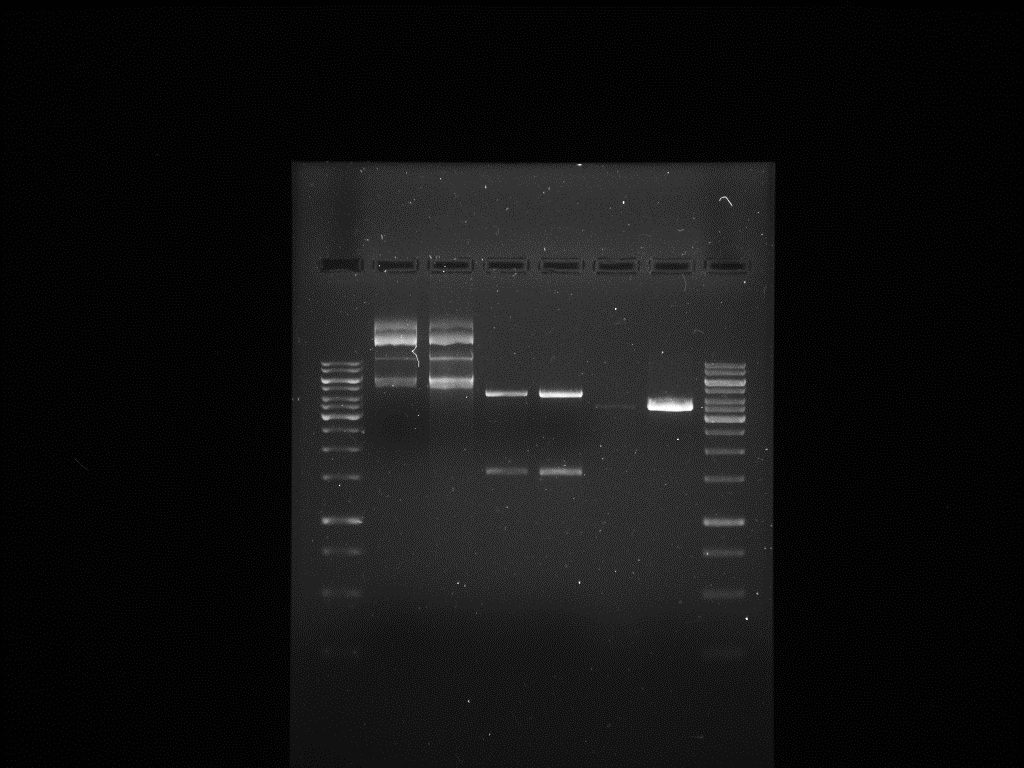


10000 bp

6000 bp

1000 bp

3000 bp

6.0 kb

Fig. S8. Recombinant construct of pGEMT-Easy_ECFP_CYP2D6_Venus 6.0 kb


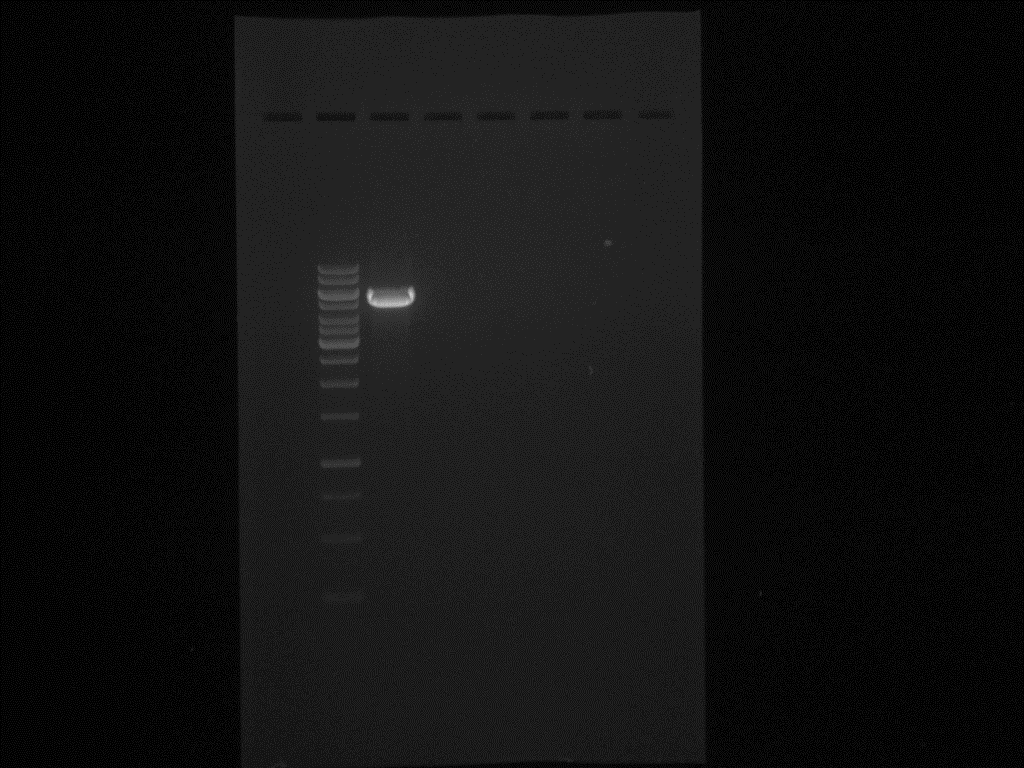


10000 bp

1000 bp

3000 bp

6000 bp

5.8 kb

Fig. S9. Recombinant construct of pRSET-B_ECFP_CYP2D6_Venus of band size 5.8 kb


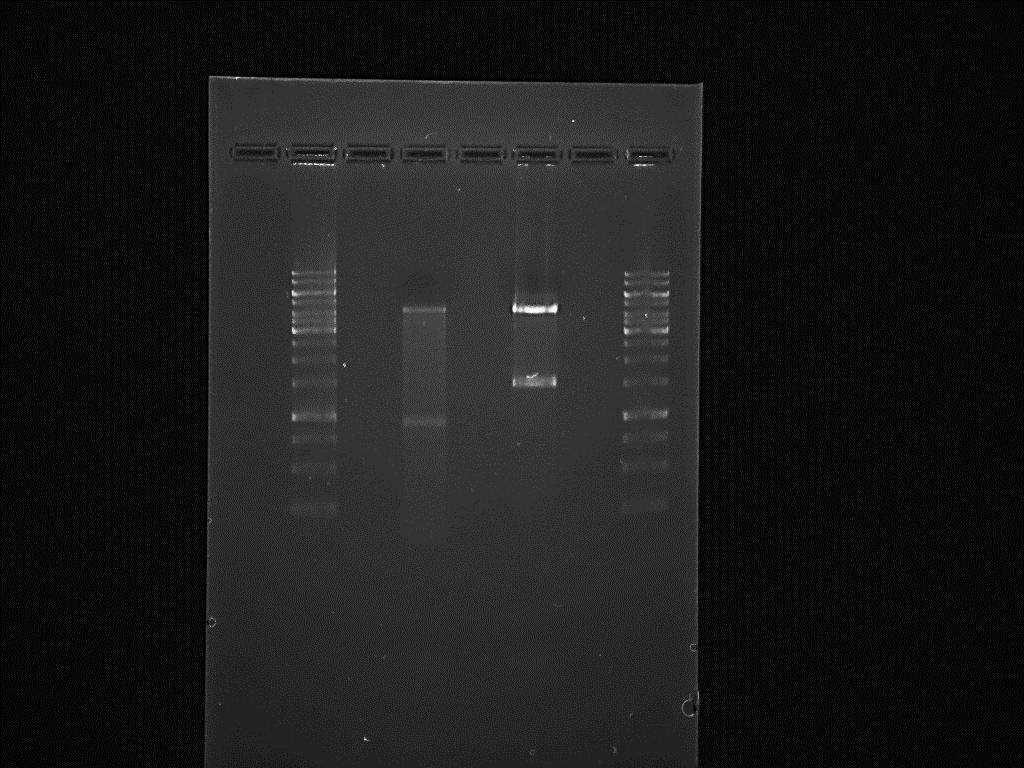


10000 bp

6000 bp

3000 bp

1000 bp

1.5 kb

4.3 kb

Fig. S10. Restriction digestion of pRSET-B_ECFP_CYP2D6_Venus with Age I yielding two band size of 4.3 kb and 1.5 kb


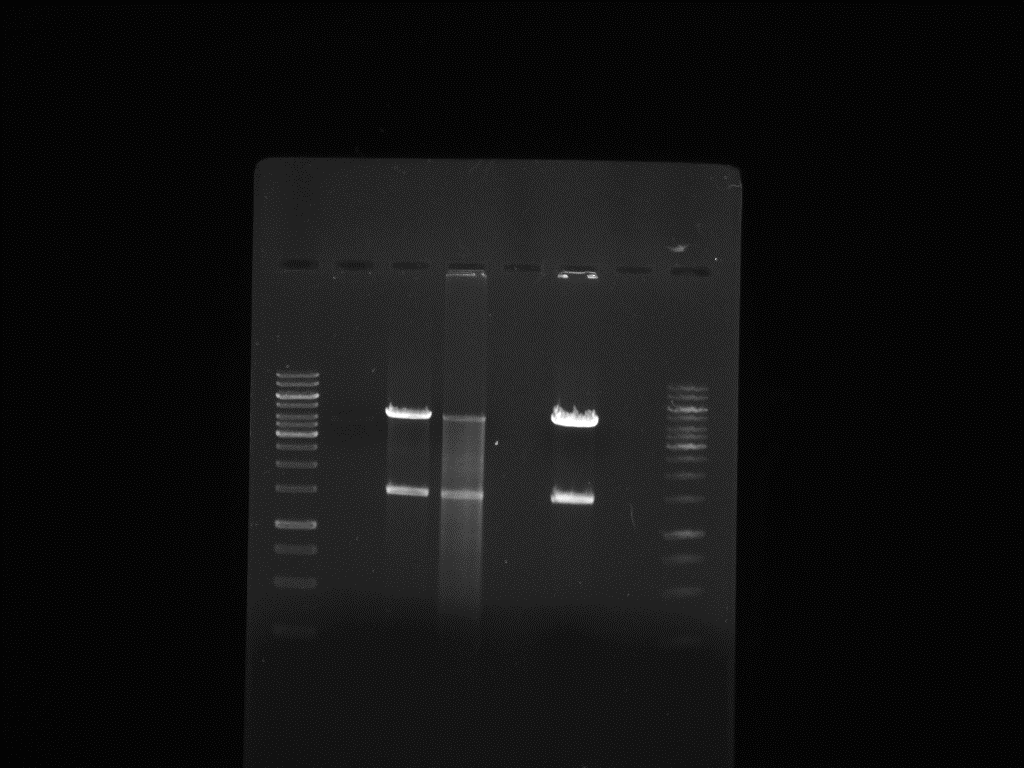


10000 bp

6000 bp

1.5 kb

4.5 kb

1000 bp

3000 bp

Fig. S11. Restriction digestion of pGEMT_ECFP_CYP2D6_Venus with Age I yielding two band size of 4.5 kb and 1.5 kb


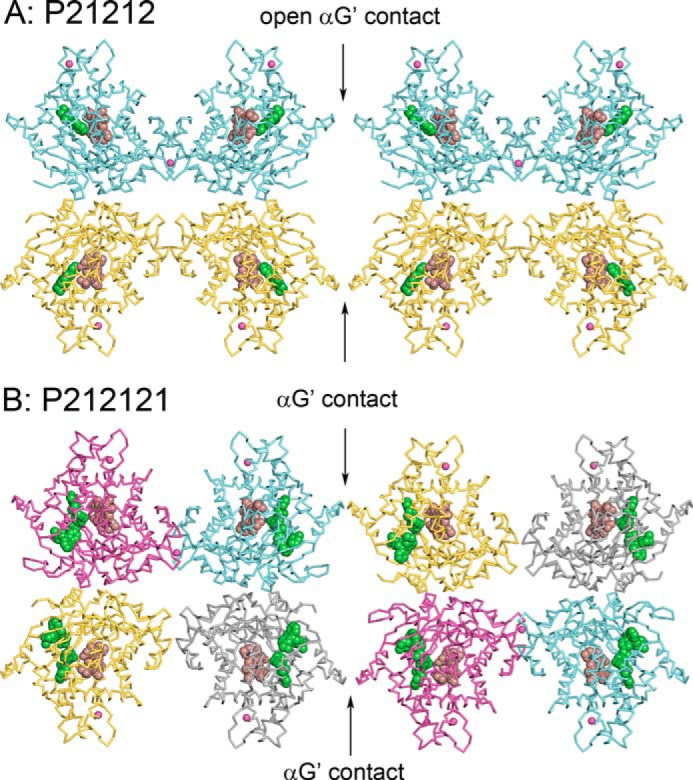


**Fig. S12.** The structural changes of CYP2D6 crystal lattice in the presence and absence of ajmalicine. (A) **The figure represents the structural changes induced in the crystal lattice of P21212 of the CYP2D6-ajmalicine complex obtained upon the displacement of thioridazine with ajmalicine in the P21212 crystal lattice (B) of CYP2D6-thioridazine complex. (reference – Wang et al., 2015)**

**GGATCCACAAGTTTGTACAAAAAAGCAGGCTATGGTGAGCAAGGGCGAGGAGCTGTTCACCGGGGTGGTGCCCATCCTGGTCGAGCTGGACGGCGACGTAAACGGCCACAAGTTCAGCGTGTCCGGCGAGGGCGAGGGCGATGCCACCTACGGCAAGCTGACCCTGAAGTTCATCTGCACCACCGGCAAGCTGCCCGTGCCCTGGCCCACCCTCGTGACCACCCTGACCTGGGGCGTGCAGTGCTTCAGCCGCTACCCCGACCACATGAAGCAGCACGACTTCTTCAAGTCCGCCATGCCCGAAGGCTACGTCCAGGAGCGCACCATCTTCTTCAAGGACGACGGCAACTACAAGACCCGCGCCGAGGTGAAGTTCGAGGGCGACACCCTGGTGAACCGCATCGAGCTGAAGGGCATCGACTTCAAGGAGGACGGCAACATCCTGGGGCACAAGCTGGAGTACAACTACATCAGCCACAACGTCTATATCACCGCCGACAAGCAGAAGAACGGCATCAAGGCCAACTTCAAGATCCGCCACAACATCGAGGACGGCAGCGTGCAGCTCGCCGACCACTACCAGCAGAACACCCCCATCGGCGACGGCCCCGTGCTGCTGCCCGACAACCACTACCTGAGCACCCAGTCCGCCCTGAGCAAAGACCCCAACGAGAAGCGCGATCACATGGTCCTGCTGGAGTTCGTGACCGCCGCCGGGATCACTCTCGGCATGGACGAGCTGTACAAGACCGGTATGGGGCTAGAAGCACTGGTGCCCCTGGCCGTGATAGTGGCCATCTTCCTGCTCCTGGTGGACCTGATGCACCGGCGCCAACGCTGGGCTGCACGCTACCCACCAGGCCCCCTGCCACTGCCCGGGCTGGGCAACCTGCTGCATGTGGACTTCCAGAACACACCATACTGCTTCGACCAGTTGCGGCGCCGCTTCGGGGACGTGTTCAGCCTGCAGCTGGCCTGGACGCCGGTGGTCGTGCTCAATGGGCTGGCGGCCGTGCGCGAGGCGCTGGTGACCCACGGCGAGGACACCGCCGACCGCCCGCCTGTGCCCATCACCCAGATCCTGGGTTTCGGGCCGCGTTCCCAAGGGGTGTTCCTGGCGCGCTATGGGCCCGCGTGGCGCGAGCAGAGGCGCTTCTCCGTCTCCACCTTGCGCAACTTGGGCCTGGGCAAGAAGTCGCTGGAGCAGTGGGTGACCGAGGAGGCCGCCTGCCTTTGTGCCGCCTTCGCCAACCACTCCGGACGCCCCTTTCGCCCCAACGGTCTCTTGGACAAAGCCGTGAGCAACGTGATCGCCTCCCTCACCTGCGGGCGCCGCTTCGAGTACGACGACCCTCGCTTCCTCAGGCTGCTGGACCTAGCTCAGGAGGGACTGAAGGAGGAGTCGGGCTTTCTGCGCGAGGTGCTGAATGCTGTCCCCGTCCTCCTGCATATCCCAGCGCTGGCTGGCAAGGTCCTACGCTTCCAAAAGGCTTTCCTGACCCAGCTGGATGAGCTGCTAACTGAGCACAGGATGACCTGGGACCCAGCCCAGCCCCCCCGAGACCTGACTGAGGCCTTCCTGGCAGAGATGGAGAAGGCCAAGGGGAACCCTGAGAGCAGCTTCAATGATGAGAACCTGTGCATAGTGGTGGCTGACCTGTTCTCTGCCGGGATGGTGACCACCTCGACCACGCTGGCCTGGGGCCTCCTGCTCATGATCCTACATCCGGATGTGCAGCGCCGTGTCCAACAGGAGATCGACGACGTGATAGGGCAGGTGCGGCGACCAGAGATGGGTGACCAGGCTCACATGCCCTACACCACTGCCGTGATTCATGAGGTGCAGCGCTTTGGGGACATCGTCCCCCTGGGTGTGACCCATATGACATCCCGTGACATCGAAGTACAGGGCTTCCGCATCCCTAAGGGAACGACACTCATCACCAACCTGTCATCGGTGCTGAAGGATGAGGCCGTCTGGGAGAAGCCCTTCCGCTTCCACCCCGAACACTTCCTGGATGCCCAGGGCCACTTTGTGAAGCCGGAGGCCTTCCTGCCTTTCTCAGCAGGCCGCCGTGCATGCCTCGGGGAGCCCCTGGCCCGCATGGAGCTCTTCCTCTTCTTCACCTCCCTGCTGCAGCACTTCAGCTTCTCGGTGCCCACTGGACAGCCCCGGCCCAGCCACCATGGTGTCTTTGCTTTCCTGGTGACCCCATCCCCCTATGAGCTTTGTGCTGTGCCCCGCACCGGTATGGTGAGCAAGGGCGAGGAGCTGTCACCGGGGTGGTGCCCATCCTGGTCGAGCTGGACGGCGACGTAAACGGCCACAAGTTCAGCGTGTCCGGCGAGGGCGAGGGCGATGCCACCTACGGCAAGCTGACCCTGAAGCTCATCTGCACCACCGGCAAGCTGCCCGTGCCCTGGCCCACCCTCGTGACCACCCTCGGCTACGGCCTCAGTGCTTCGCCCGCTACCCCGACCACATGAAGCAGCACGACTTCTTCAAGTCCGCCATCCCGAAGGCTACGTCCAGGAGCGCACCATCTTCTTCAAGGACGACGGCAACTACAAGACCCGCGCCGAGGTGAAGTTCGAGGGCGACACCCTGGTGAACCGCATCGAGCTGAAGGGCATCGACTTCAAGGAGGACGGCAACATCCTGGGGCACAAGCTGGAGTACAACTACAACAGCCACGACTTCAAGGAGGACGGCAACATCCTGGGGCACAAGCTGGAGTACAACTACAACAGCCGCACAACATCGAGGACGGCGGCGTGCAGCTCGCCGACCACTACCAGCAGAACACCCCCATCGGCGACGGCCCCGTGCTGCTGCCCGACAACCACTACCTGAGCTACCAGTCCAAGCTGAGCAAAGACCCCAACGAGAAGCGCGATCACATGGTCCTGCTGGAGTTCGTGACCGCCGCCGGGATCACTCTCGGCATGGACGAGCTGTACAAACCCAGCTTTCTTGTACAAAGTGGTAAGCCT**

**ECFP** – denoted in cyan color; **CYP2D6** – denoted in pink color; **Venus** – denoted in yellow color; ***att*B1 site** – denoted in grey color; ***att*B2 site** – denoted in blue color; ***BamH*I** **site** – denoted in red color; ***Age*I** – denoted in green color; ***Hind*III**- denoted in black color

Fig. S13. Nucleotide sequence of ECFP_CYP2D6_Venus cloned in pRSET-B
